# High-Speed Video System for Micro-Expression Detection and Recognition

**DOI:** 10.3390/s17122913

**Published:** 2017-12-14

**Authors:** Diana Borza, Radu Danescu, Razvan Itu, Adrian Sergiu Darabant

**Affiliations:** 1 Computer Science Department, Technical University of Cluj-Napoca, 28 Memorandumului Street, 400114 Cluj Napoca, Romania; radu.danescu@cs.utcluj.ro (R.D.); razvan.itu@cs.utcluj.ro (R.I.); 2 Computer Science Department, Babes Bolyai University, 58-60 Teodor Mihali Street, C333, 400591 Cluj Napoca, Romania; adrian.darabant@tvarita.ro

**Keywords:** micro-expression spotting, micro-expression recognition, affective computing, facial expression recognition, difference images

## Abstract

Micro-expressions play an essential part in understanding non-verbal communication and deceit detection. They are involuntary, brief facial movements that are shown when a person is trying to conceal something. Automatic analysis of micro-expression is challenging due to their low amplitude and to their short duration (they occur as fast as 1/15 to 1/25 of a second). We propose a fully micro-expression analysis system consisting of a high-speed image acquisition setup and a software framework which can detect the frames when the micro-expressions occurred as well as determine the type of the emerged expression. The detection and classification methods use fast and simple motion descriptors based on absolute image differences. The recognition module it only involves the computation of several 2D Gaussian probabilities. The software framework was tested on two publicly available high speed micro-expression databases and the whole system was used to acquire new data. The experiments we performed show that our solution outperforms state of the art works which use more complex and computationally intensive descriptors.

## 1. Introduction

Micro-expressions (ME) are fast, involuntary facial movements that last only a fraction of a second and they occur either as a form of suppression (deliberate concealment) or repression (unconscious concealment). Micro-expressions (MEs) were first discovered by Haggard and Isaacs [1]: by reviewing psychotherapeutic interviews, they observed “micro-momentary” facial expressions and explained them as a result of repression. They also stated that these movements could not be recognized in real time. 

Independently, Eckman’s [2] studies on deceit revealed the existence of the same subtle facial movements; while reviewing videos of depressed patients that falsely claimed to be happy and later tried to commit suicide, he observed that the patients had brief moments in which they displayed strong negative feelings through micro-expressions. Since then, micro-expressions have been considered reliable sources of deceit detection and various ME books [3] and training programs [4] have been created to help people understand others and enhance their relationships. The Facial Action Coding system is generally used to classify facial expressions based on the movement of individual facial muscles or groups of muscles (action units).

Although there are still some critical reviews [5], it is generally accepted that MEs can express the seven universal emotions: anger, fear, sadness, disgust, surprise, contempt, and happiness [2]. Three key moments can be distinguished during a micro-expression: the onset (the moment when the ME starts to appear), the apex (the moment when the ME achieves its maximum amplitude) and the offset (the moment when the ME faded out).

Automatic analysis of facial expressions has been largely studied in the last decades [6], but ME analysis has not been extensively explored. Automatic analysis of MEs must address several challenges. First, MEs are fast and subtle facial movements and their analysis requires accurate and robust motion descriptors. In addition, due to their short duration, ME sequences must be captured using high speed capturing devices. Finally, as MEs are involuntary movements that occur only in high-stake situations the training and test data is hard to gather.

Several ME databases were published in the literature. The Polikovsky dataset [7] contains video sequences of 10 subjects captured at a frame rate of 200 fps and annotated with the action-units. Its main drawback is that the expressions are posed: the subjects were asked to express the seven universal facial expressions with low intensity and rapidly return to the neutral state. However, these posed expressions have different dynamics than the genuine ones [8].

Other databases contain authentic ME [9,10,11,12]. To induce genuine emotions, the subjects were asked to try to hide their feelings while watching videos with high emotional valence. If failed to do so, the participants were given a penalty and were asked to fill in a boring and long questionnaire [9]. By using this setup, a high-stake situation was created in which MEs are more likely to occur. Video clips were used as stimuli because they contain both visual and audio information (as opposed to images) and due to their longer duration, inhibition of emotions is more difficult. From a theoretical perspective, videos stimuli are more suitable interactive real-world situations, because it is easier to capture stable face data and to spot and recognize the micro-expressions (even for a human operator). 

The SMIC database [9,10] was collected at a temporal resolution of 100 fps and it contains 164 MEs of 16 subjects. The sequences are annotated with three emotion labels: positive, negative and surprise. In addition, for 10 subjects video recordings captured with both visual and near-infrared regular (25 fps) cameras are included in the database. Recently, a new version of the database, SMIC-E, was published, which also contains some non-expression frames before and after the labeled micro-frames. The average video duration in this extended database is 5.9 s and it can be used for the ME detection task. CASME II [12] database contains 247 micro-expression videos annotated with action units and seven emotion labels. The sequences were captured in a highly controlled laboratory environment at 200 fps. Thirty-five subjects participated in the study, with a mean and standard deviation of age of 22.03 and 1.60 years, respectively. 

Recently, the SASE-FE [13] database was developed for the analysis of genuine vs. deceptive emotional displays from high resolution, high frame-rate video sequences. The dataset contains 643 videos of 50 different subjects, recorded at a frame rate of 100 fps with a high-resolution GoPro-Hero camera. To elicit the emotions, participants were asked to watch videos with high emotional valance from YouTube. At the beginning of a video sequence, the subjects started their portrayals with a neutral expression, then they watched a video to elicit a genuine facial expression and, finally, they were asked to act/pose another facial expression of emotion (the opposite of the former). The average length of a video sequence is 4 s. ME analysis involves two different tasks: detection and recognition. ME detection refers to the process of determining if a subtle expression occurred at a given frame and establishing the key moments of the ME (onset, apex and offset). On the other hand, ME recognition involves establishing the exact type of the expression. It is generally accepted that the MEs are universal and correspond to the seven basic emotions; however, because they are involuntary and un-posed, and spontaneous data are hard to gather, a more simplified taxonomy, with only three classes (positive, negative and surprise) is often used. 

Automatic recognition of MEs follows the classical stages of the pattern recognition: *region proposal*, *feature extraction* and *learning*. In the region proposal step, several relevant zones on the face are selected to be analyzed in the next steps. Four approaches are usually used: (1) splitting the face into free-form regions; (2) geometrically splitting the entire face into equally sized cells [14,15,16]; (3) selecting some rectangular cells around the most prominent facial features [17]; and (4) analyzing the entire face as a whole [18]. In the feature extraction step, reliable motion descriptors must be computed to numerically express the motion that occurred during a ME. Multiple descriptors were proposed: Local Binary Patterns in Three Orthogonal Planes (LBP-TOP), optical flow, optical strain [15,16] or histograms of 3D gradients [17]. Finally, a machine learning algorithm is trained to decide on the exact type of ME that occurred.

In [17] the face is divided into 12 rectangular regions around the most prominent parts and the motion information from each region is recognized based on 3D gradient orientation histograms. Thirteen types of MEs on the Polikovsky’s dataset are recognized as well as their three key moments (onset, apex and offset). Another motion descriptor that is used in the detection and recognition of ME is the optical strain [15,16], an extensive version of optical flow “that is capable of quantifying subtle changes on faces and representing the minute facial motion intensities at the pixel level”. In [14], the optical strain information is used as weight factor to LBP-TOP features and a support vector machine classifier (SVM) is used to detect and recognize MEs in high speed videos. The work presented in [15] tackles both the problem of ME detection and recognition. For the detection part, three facial points (eye inner corners and tip of the nose) are detected and tracked in the input video and the face is split into 36 blocks based on these points. Next, two types of features are extracted: Histograms of Oriented Optical Flow (HOOF) and LBP. Finally, the MEs are spotted by thresholding histogram differences. For the recognition part, the faces are aligned and Eulerian motion magnification [19] is applied to enhance facial motion. Next, several features are extracted (LBP-TOP, Histograms of Oriented Gradients on Three Orthogonal Planes and Histogram of Image Gradient Orientation on Three Orthogonal Planes) and a linear SVM is used to recognize the ME type.

Recently, since deep learning achieved impressive results in various classification tasks, nearly all the computer vision related tasks have been reinterpreted from a “deep” perspective. Some works propose deep learning techniques for ME detection [19] and recognition [20]. In [19], a deep neural network was only used to detect 68 landmarks on the face and next the onset, apex and offset frames were classified by a SVM based on histograms of oriented optical flow (HOOF). A fully deep approach is used in [20]: a convolutional neural network (CNN) was trained using the frames from the beginning of each ME sequence and the onset, apex and offset frames. Next, the convolutional layers of the trained network were combined with a long-short-term-memory recurrent neural network, by connecting its input to the first fully connected layer of the CNN. 

In this paper, we propose a novel ME detection and classification technique, with the following original contributions:

- The development of an intelligent ME image acquisition system which does not require rigid position of the subject, and uses two frame acquisition setups: (a) normal angle, full frame, normal frame-rate image acquisition for locating the subject’s face; and (b) a narrow angle, region of interest, high speed acquisition for detection and classification of MEs.
- The analysis of 10 square regions on the face that roughly correspond to the muscles of facial expressions to detect and recognize spontaneous MEs in high speed video sequences.
- The development of simple and effective motion descriptors based on simple image differences. We define the motion magnitude image as the absolute difference between the current frame (a potential apex frame) and the frame half the ME duration ahead (a potential onset frame) divided by the absolute difference between the current frame and a previous neighbor frame (three frames ahead in our experiments). The first difference image describes the motion variation, while the latter one is used as a normalization factor to filter out noise.
- The development of a micro-expression detection module based on the magnitude (the average value within each selected facial cell) of the movement magnitude images using an ensemble learning algorithm. The raw classifier predictions are post-processed, based on the assumption that there should be an agglomeration of positive predictions between the onset and offset ME frames, in order to ensure the robustness of the ME spotting algorithm.
- The development of a ME recognition module which uses the movement magnitude image to determine the motion direction within each facial cell: the relative displacement of the weighted centroid of each cell is tracked during several frames of the ME interval. The training part involves only the fitting a 2D Gaussian function on the relative center displacements of the samples from the training set.
- Finally, the detection and recognition module are integrated into a fully automatic ME analysis framework.

The remainder of this work is organized as follows: in Section 2, we present the outline of the ME analysis framework and the proposed approach is detailed in Section 3. The experimental results are discussed in Section 4. Finally, Section 5 concludes this work.

## 2. System Setup

We designed a physical system to elicit and capture micro-expressions: we used a high speed Ximea camera [21] connected to a regular PC; the PC displays videos with high emotional valence to the user and records his/her reaction to these stimuli using the high temporal resolution camera. The system uses a high speed MQ022CG-CM USB3.0 Ximea camera equipped with a Fujinon 9 mm lens (ensuring a horizontal field of view of 64° and a vertical field of view of 37°), able to capture 2048 × 1088 video frames at a maximum rate of 170 fps. The camera can be configured to use a ROI-based image acquisition and thus allowing higher frame rates.

We used the computer clock to get the timestamp of each frame. The transfer time from the camera to the PC requires a fixed amount of time for the same ROI. We are interested in the relative difference between the frames and not the exact timestamp of the frame. In practice, a reasonable bandwidth for the USB 3 controllers is around 3.2 Gbit/s (0.4 GB/s or 400 MB/s) and in our experiments the maximum data flow produced by the camera was 151 MB/s. Thus, the bandwidth taken on one camera alone on the controller does not produce any bottlenecks on the controller.

**Figure 1:**
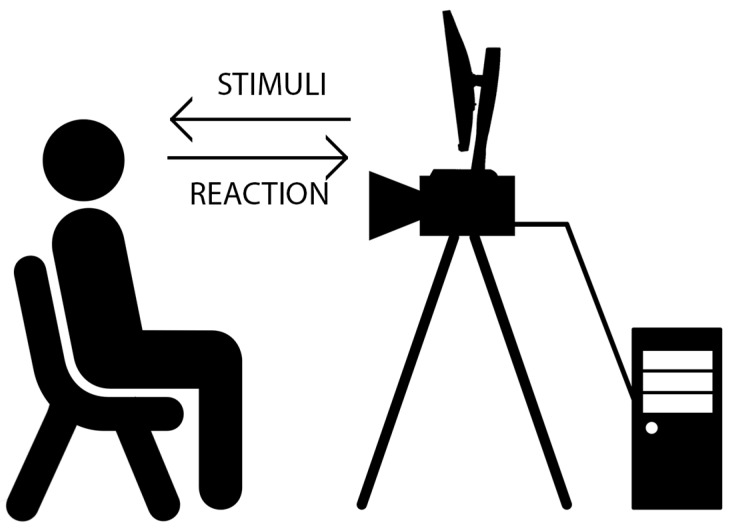
High-speed video acquisition and analysis process.

The basic use-case of the proposed system is the following one (Figure 1): the user is asked to sit in view of the camera and will be subjected to stimuli that may cause emotional response (for example, asked to watch selected videos).

**Figure 2:**
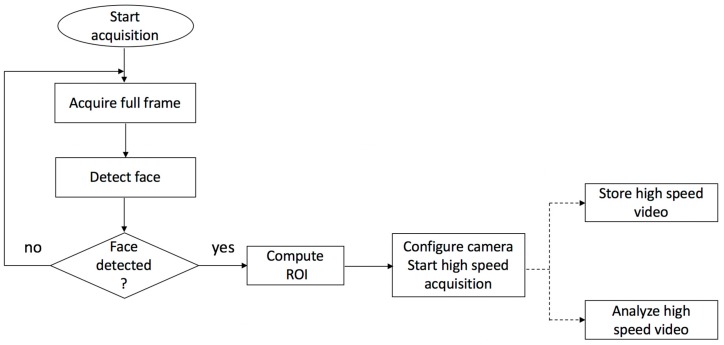
High-speed video acquisition and analysis process.

The system starts to acquire images at full frame (2048 × 1088) using a 30 fps. The image processing host computer automatically detects the user’s face using a publicly available face detection library [22]. After the face is detected, a region of interest is automatically established such that it will include the detected face and a significant safety area around this face (the width and height of the ROI are 75% larger than the dimensions of the detected face). The camera is configured to use a ROI-based image acquisition method using the detected ROI which significantly reduces the data amount to be transferred via USB and stored in the computer’s memory, thus allowing high-speed video capture (more than 110 fps). The actual frame rate depends not only on the data amount, but also on the exposure time, which is not influenced by the ROI size. The flowchart of this process is depicted in Figure 2.

This value was chosen heuristically. A smaller region of interest allows a higher framerate; on the other hand, by allowing a wider region of interest, the system has a higher tolerance towards head movement, but the frame rate is decreased. The goal is to obtain an equilibrium between these two conditions such that the user is allowed a natural, unrestrictive head-movement and the camera can capture high-speed video sequences. In addition, resetting the camera region requires some amount of time, which implies a loss of framerate. The face detection is repeated from *τ* to *τ* frames, and, only if necessary, the region of interest is reset on the camera.

The system can be used to either capture and store the high-speed video-sequences or to online analyze the video stream (detect and recognize MEs).

**Figure 3:**
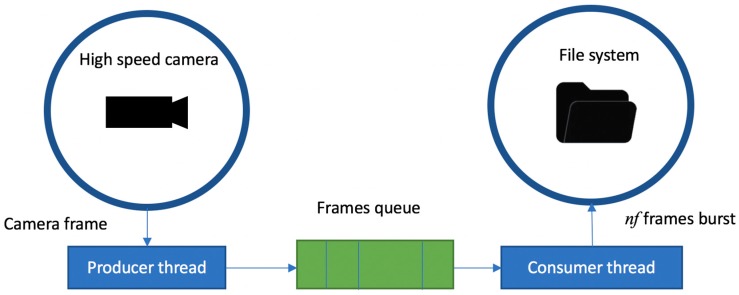
High-speed video capturing and storing process.

To save the high-speed video frames, we used the well-known producer-consumer synchronization mechanism (Figure 3). The producer thread reads the high-speed video frames from the Ximea camera and saves them to the shared data queue, while the consumer thread reads the frames from the queue and saves them to the physical drive. The consumer thread needs to constantly interact with the file system, which is a time-consuming process, while the producer thread reads the frames from the camera with a high temporal resolution. To avoid out-of-memory errors, the frames and their corresponding timestamps are saved on the hard-drive in bursts of *nf* = 100 frames, directly in binary format using low level system calls.

Once the recording is finished, the binary files are processed and the frames are saved on the hard drive in .bmp format with the time stamp on their name.

## 3. Proposed Solution for Micro-Expression Detection and Classification

### 3.1. Outline of the Micro-Expression Detection and Classification Process

**Figure 4:**
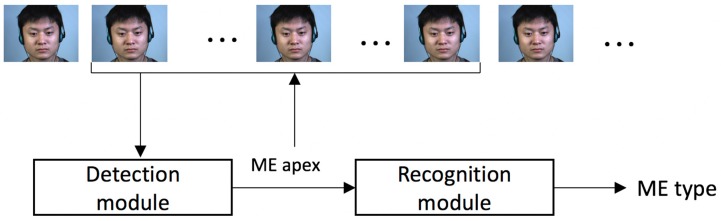
Outline of the proposed solution. The detection module determines if a ME occurred and its corresponding apex frame. The recognition module further analyses the frames and computes the type of the ME that occurred: negative, positive or surprise (raw frames from CASME II database [12] (©Xiaolan Fu)).

We propose a framework for ME detection and recognition based on simple motion descriptors. The outline of the proposed solution is depicted in Figure 4.

The framework takes as input full video clips and computes the apex positions as well as the types of the MEs that occurred. First, the detection module determines the moment (the apex frame) when a ME occurred based on the motion magnitude variation across the video frames. The detected apex locations are fed to the recognition module, which uses only the frames around the apex position to determine the type of the ME (positive, negative or surprise). 

### 3.2. Facial Landmarks Detection and Cell Selection

**Figure 5:**
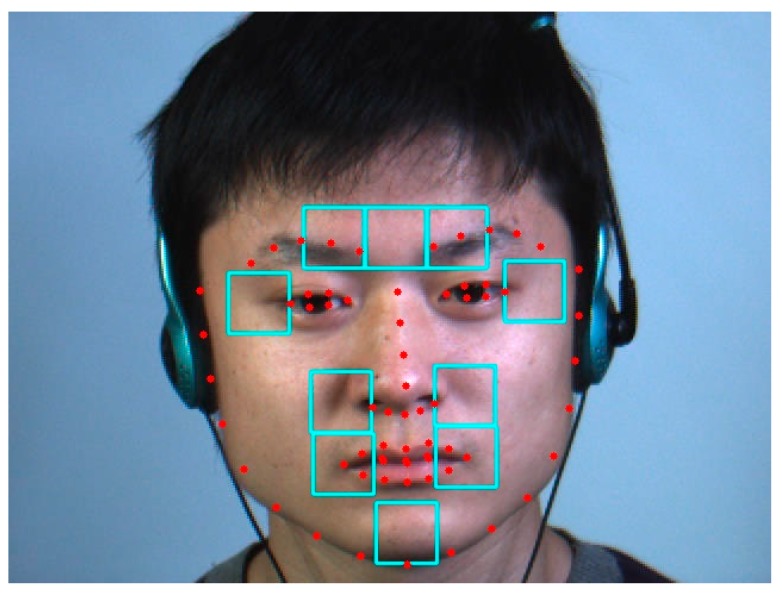
Regions of interest selection. The positions of the 68 facial landmarks are depicted in red circles and the 10 facial regions used to analyze the MEs are drawn with cyan rectangles (raw frames from CASME II database [12] (©Xiaolan Fu)).

A general off the shelf facial landmark detector [23,24] is used to detect 68 landmarks on the face. Based on the position of these landmarks, we defined 10 regions on the face that roughly correspond to the position of the muscles of facial expressions, as shown in Figure 5.

The three upper cells correspond to the left frontalis, procerus and right frontalis muscles, respectively. The two cells around the eyes overlap the orbicularis oculi muscles. The four cells around the nostrils and mouth area are related to the orbicularis oris and zygomatics muscles. Finally, the cell in the chin area corresponds to the mentalis muscle [25]. The width of a cell was heuristically set to half the mouth width. 

Based on the orientation determined by the off-the-shelf detector, we correct the small face orientations with normalization; more specifically, the face is rotated such that the roll angle becomes 0.

### 3.3. Micro-Expression Detection

**Figure 6:**
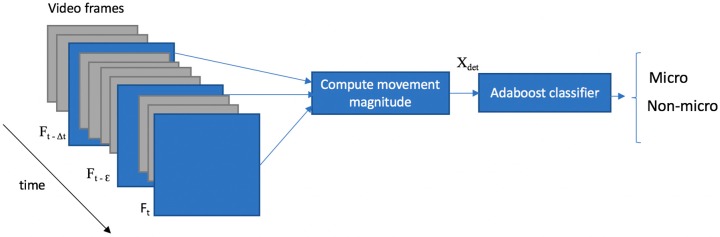
ME detection framework. The frames that are analyzed by the ME detection module at time *t* (F*_t_*_−*τ*/2_, F*_t_*_−*ε*_ and F*_t_*) are depicted in blue, while the other frames from the micro-expression sequence are depicted in gray.

The detection module relies on the magnitude of the movement that occurs across the high-speed video frames computed by simple absolute image differences. The motion information is extracted from each frame and Adaboost [26,27] algorithm is used to decide if a frame belongs to the ME or the non-ME class. The outline of the detection module is depicted in Figure 6.

Let *τ* denote the average ME duration in frames; we computed this value to 65 frames for CASME II dataset and 37 for the SMIC-E dataset.

As the aim of the detection module is to find the apex frames, we consider the absolute image difference between the current frame *t* (a potential apex frame) and the previous frame at a distance *τ*/2 (a potential ME start frame). However, as the facial movements that occur during MEs have very low intensity, we also introduce a normalization factor to distinguish the ME motion from the noise caused by illumination conditions or the capturing devices. The frame *t* − *ε* (*ε* = 3 in our experiments) is used as a normalization factor. Because the video sequences are captured with high speed cameras, no facial movement should occur in 0.03 s (value computed for a 100 fps temporal resolution).

Finally, the motion magnitude variation is computed as the absolute difference between the frame *t* and *t − τ*/2, normalized with the absolute difference between *t − ε* (Equation (1)).

(1) $$MM_{i}=\frac{\left|frame_{t}-frame_{t-\frac{\tau }{2}}\right|+1}{\left|frame_{t}-frame_{t-\varepsilon }\right|+1}$$

**Figure 7:**
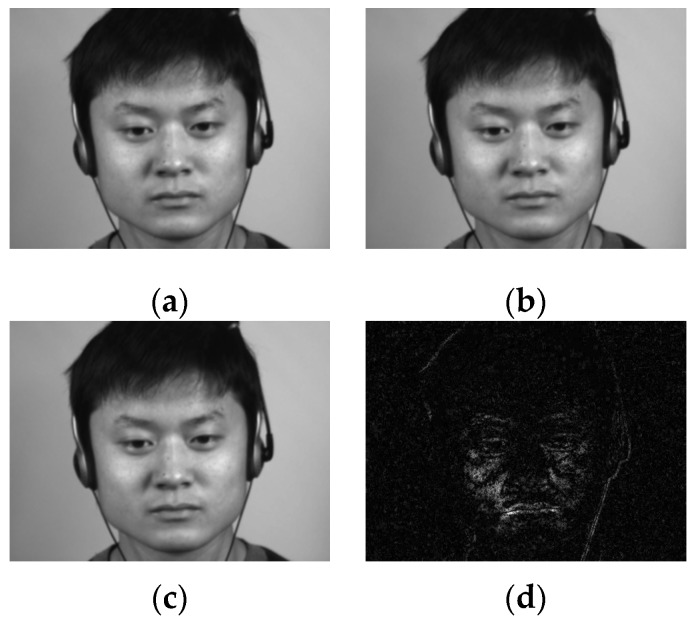
Frame differences for ME analysis: (**a**) frame *f_t_*_−*τ/*2_ (the potential onset frame); (**b**) frame *f_t_*_−*ε*_ (used for normalization); (**c**) current frame *f_t_* (the potential apex frame); and (**d**) movement magnitude variation (raw frames from CASME II database [12] (©Xiaolan Fu)).

Figure 7 shows the frame difference images that are used for the ME detection task.

**Figure 8:**
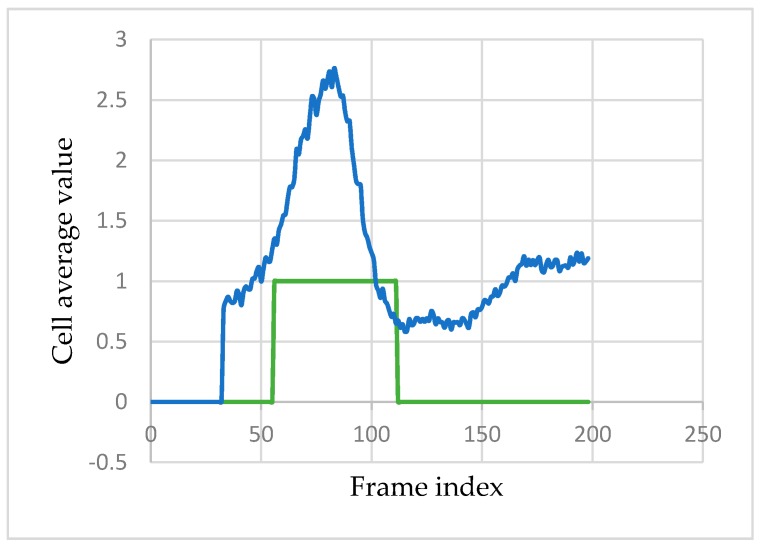
Middle eyebrow cell *µ_c_* (*MM_i_*) variation across a ME sequence. The ground truth labeling of the ME sequence is marked with a green step function, and the value of *µ_c_* (*MM_i_*) is depicted in blue. The first *τ/2* are ignored (*µ_c_* (*MM_i_*) is set to 0) as the MM image cannot be computed for frames *t,* where *t < τ* /2. On the x-axis the frame index in the MEs sequence is represented and on the y-axis the average value of the movement magnitude variation within the cell is plotted.

However, only 10 regions on the face are analyzed (Section 3.1). Therefore, for each cell *c*, the average value of the movement magnitude image (*µ_c_* (*MM_i_*)) within that region of interest is computed. For example, Figure 8 shows the variation of *µ_c_* (*MM_i_*) for the middle eyebrow cell during a ME sequence.

To extract the feature vector for the classification of a frame, all cells are considered. For a new test frame *i*, a window of size *τ* is centered in the current frame. For each one of the 10 facial cells (*c*), the minimum and maximum value of the *µ_c_* (*MM_i_*) in the interval [*i* − *τ* /2, *i* + *τ* /2] is extracted. In other words, the extracted feature vector for each frame can be expressed as:

(2) $$X_{det_{i}}=[\min(w_{c_{0}}),\;\max(w_{c_{0}}),\;\min(w_{c_{1}}),\;\max(w_{c_{1}}),\;\ldots ,\;\min(w_{c_{9}}),\;\max(w_{c_{9}})]$$

where $w_{c_{i}}$ is the variation of the *µ_c_* (*MM_i_*) values for the cell $c_{i}$ in the time window centered in the current frame:

(3) $$w_{c_{i}}=\left[\mu_{c}\left(MM_{t-\frac{\tau }{2}}\right),\mu_{c}\left(MM_{t-\frac{\tau }{2}+1}\right),\;\ldots ,\;\mu_{c}\left(MM_{t+\frac{\tau }{2}}\right)\right]$$

To label the training images into micro and non-micro frames, the following rule is used:

- If *t* ∈ [0, *t_apex_* − *δ* ∙ *τ*] or *t* ∈ [*t_apex_ +* *δ* *∙* *τ*, *seqLen*], then the frame *t* is labeled as non-ME frame (neutral frame or macro-expression). *seqLen* is the video sequence length in frames.
- If *t* ∈ (*t_apex_* − *δ ∙ τ*, *t_apex_ + δ ∙ τ*), then frame *t* is considered a ME frame, where the factor *δ* is set heuristically to 0.25. In other words, we define an interval of size half the average ME centered in the apex frame which will contain the frames labeled as ME frames

However, the training set is highly unbalanced: there are many more non-ME frames than ME frames. For training, we use all the available ME frames and we randomly select an equal number of non-ME frames.

Finally, the feature vector is inputted to Adaboost algorithm to determine the type of each frame. Adaboost is a meta-estimator classifier which uses a set of “weak” learners or estimators which are combined into a weighted sum in order to boost the classification performance. At each iteration of the learning algorithm, the weak estimators are adjusted such that they focus on the instances previously misclassified by the algorithm. We have used 35 weak estimators (Decision Tree Classifiers).

Using the algorithm and the labeling described above, one would expect the classifier to predict multiple micro-frames around the real apex. Therefore, the response of the classifier is further post-processed in order to filter out false positives and to merge the positive responses which belong to the same ME. First all the disjunctive ME intervals are detected and the intervals that are too close to each other are merged together. Finally, the size of each interval is examined, and the intervals that are too short are ruled out (Algorithm 1). The apex frames are set to the middle of each predicted ME interval.

**Algorithm 1.** ME detection post processing.Params: minMicroSz: the minimum size in frames of a ME (*τ*/4 in our experiments) maxDist: the maximum distance between two clusters to be merged (2∙*τ* in our experiments)1: Find the predicted and disjunctive ME intervals: *I* = {(*s*_0_, *e*_0_), (*s*_1_, *e*_1_), …, (*s_n_*, *e_n_*)} 2: doMerge ← True 3: while doMerge do 4: doMerge ← False 5: for *i* = 1 to *len(I)* do 6: *m*_1_ <- (*e_i_*_-1 _– *s_i_*_-1_) 7: *m*_2_ <- (*e_i_* – *s_i_*) 8: if (*m*_2_ – *m*_1 _) < maxDist then 9: merge(*I_i_*, *I_i_*_-1_) 10: doMerge ← True 11: break 12: end if 13: end for 14: end while 15: for *i* = 1 to *len(I)* do 16: if (*e_i_* – *s_i_*) < minMicroSz then 17: remove(*I_i_*) 18: end if 19: end for

### 3.4. Micro-Expression Recognition

**Figure 9:**
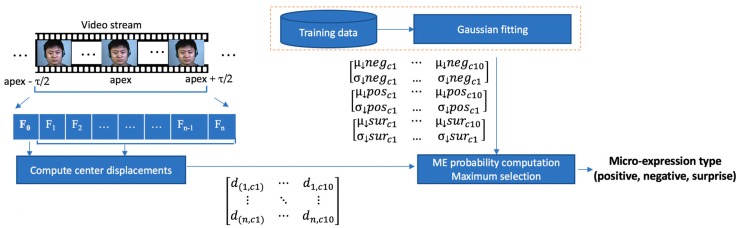
ME recognition module.

The flow of the ME recognition module is depicted in Figure 9. The features used to recognize the ME type is the relative center displacement of the *movement magnitude* image within each facial cell. During the training phase, a 2D Gaussian is fit to the data for each ME type (positive, negative and surprise). For the test phase, to decide the type of a new ME sequence, we simply compute and multiply the probabilities of the cell movements to belong to the ME classes.

**Figure 10:**
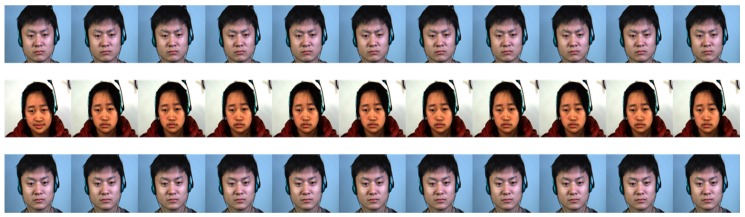
Example of the 11 frames used to recognize the type of the ME. First row: an example of a negative ME sequence, second row: an example of a positive ME sequence, third row: an example of a surprise ME sequence (raw frames from CASME II database [12] (©Xiaolan Fu)).

A window of size *τ* (the average duration of a ME) frames is centered in the apex frame and the motion direction information is used to recognize the type of the ME. To make the algorithm invariant to the frame rate of the capturing device, this interval is re-sampled into *n* = 11 video frames: {*F*_0_, *F*_1_, *F*_2_, …, *F_n_*}. The value of *n* was determined through trial and error experiments. An example of the *n* frames used to recognize a ME type is depicted in Figure 10.

To extract the motion information, we propose a simple descriptor based on the movement magnitude image MM. For each cell, we compute the weighted centroid position based on the intensity of each pixel of the movement magnitude image:

(4) $$c_{x}=\frac{1}{\mu_{MM}}\overset{c_{M}}{\underset{x=c_{s}}{\sum }}\overset{r_{M}}{\underset{y=r_{s}}{\sum }}x\cdot MM\left(x,y\right)$$

(5) $$c_{y}=\frac{1}{\mu_{MM}}\overset{c_{M}}{\underset{x=c_{s}}{\sum }}\overset{r_{M}}{\underset{y=r_{s}}{\sum }}y\cdot MM\left(x,y\right)$$

where *µ_MM_* represents the sum of the motion magnitude pixels within the cell; *MM(x, y)* is the value of the motion magnitude image at the position (*x*, *y*); and *c_s_*, *r_s_*, *c_M_*, and *r_M_* define the bounding rectangle of the cell. 

The position of the center (*cx*_0_, *cy*_0_) of the first frame *F*_0_ is considered the baseline center position of the cell in the neutral case. Next, we compute the difference between the weighted center of each frame *F_i_, i* ∈ 1, *n* and the baseline position: (*cx*_0_, *cy*_0_). These displacements constitute the feature vector used in the ME recognition procedure:

(6) $$X_{i}=\left[\left({\mathrm{cx}}_{1}–{\mathrm{cx}}_{0},\;{\mathrm{cy}}_{1}–{\mathrm{cy}}_{0}\right),\;\left({\mathrm{cx}}_{2}–{\mathrm{cx}}_{0},\;{\mathrm{cy}}_{2}–{\mathrm{cy}}_{0}\right)\ldots \;,\;\left({\mathrm{cx}}_{n}–{\mathrm{cx}}_{0},\;{\mathrm{cy}}_{n}–{\mathrm{cy}}_{0}\right)\right]$$

where (*cx_i_*, *cy_i_*) is the position of the weighted magnitude center within the current cell of frame *F_i_*.

The main advantages of the proposed descriptor are that it is easy and fast to compute and it matches the process itself: the descriptor has a direct connection with the muscular action involved in MEs. Ideally, the motion descriptor to be used should be dense optical flow, but its main disadvantages are that it is slow to compute, it is computationally expensive and, for the problem of MEs detection, it is not very correctly determined because the face area is mostly homogeneous and does not have many detectable features. Moreover, the motion amplitude is very low, so it is almost impossible to detect it at a pixel level.

**Figure 11:**
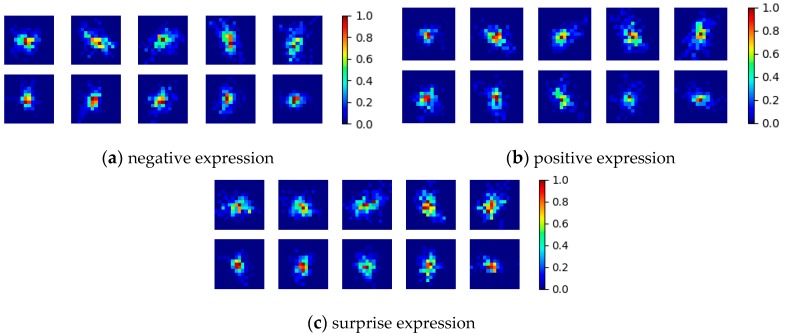
The relative center displacement for each one of the ten cells visualized as a color map: (**a**) relative center displacement for the negative expressions; (**b**) relative center displacement for the positive expressions; and (**c**) relative center displacement for the surprise expressions.

By splitting the face into regions which roughly overlap with the facial muscles of the expression, we expect that the movement from each region has a single, predominant direction. Moreover, the computation of the relative displacement of the weighted centroid of each cell statistically determines the global motion within that cell, even if at pixel level this is very hard to compute. During the training phase, the above described feature vector *X_i_* is extracted from each ME vector (Figure 11), and a 2D Gaussian function is fit on this data. In other words, for each ME type (positive, negative and surprise), we compute the mean and covariance of the relative center displacements for each cell. For each cell, the following mean vectors and covariance matrices are computed: *µ_sur_*, *∑_sur_*, *µ_pos_*, *∑_pos_*, *µ_neg_*, and *∑_neg_*.

To determine the type of a new ME, the feature vector *X* is extracted from the detected frames. Then, for each cell, we compute the probability of the in-cell movement to belong to ME classes. For this, we use the multi-variate normal distribution function:

(7) $$p\left(x,\;\mu ,\sum^{\text{​}}\right)=\frac{1}{\sqrt{{\left(2\pi \right)}^{k}\det\left(\sum \right)}}e^{-\frac{1}{2}{\left(x-\mu \right)}^{T}\;\sum^{-1}\left(x-\mu \right)}$$

Finally, the probability of the sequence to belong to each ME class (*p_sur_*, *p_neg_* and *p_pos_*) is computed by multiplying the probabilities of all the cells. Finally, the type of the ME is selected as the maximum of *p_sur_*, *p_neg_* and *p_pos_*:

(8) $$ME=\underset{e\in \left\{pos,\;neg,\;sur\right\}}{\mathrm{argmax}}\underset{c\;\epsilon \;cells}{\sum }\log p_{c}\left(x_{c},\;\mu_{e_{c}},\sum_{e_{c}}\right)$$

where $p_{c}\left(x,\;\mu_{e_{c}},\;\sum_{e_{c}}\right)$ is the probability of the displacement of the feature vector from cell *c* to belong to the expression *e.* The probabilities are logarithmized to ensure the numerical stability of the result.

## 4. Results

In this section, we report the results of the proposed ME detection and recognition framework on the two publicly available high speed ME databases CASME II and SMIC. We used the raw video frames for classification. For all experiments, “leave one subject out” cross-validation was used: only subjects which were not used in the training step are used to test the performance of the algorithm. This evaluation protocol is more challenging than the “leave one sample out” cross-validation methodology: in the latter evaluation methodology, some video sequences are used in the testing phase, even if other samples of the same subject were used in the training phase. Finally, we present a comparison with the state of the art.

### 4.1. Micro-Expression Detection

The ME detection part was tested on the CASME II dataset and on the high-speed SMIC-E dataset (a more recent version of SMIC dataset that includes longer video sequences). The average duration of a video clip is 1.3 s and 5.9 s for the CASME II and SMIC-E databases, respectively. If the ME sequence is too short, the sequence is ignored (we ignored three sequences from the CASME II database).

The confusion matrices for the detection module are illustrated in Table 1. In the table below the metrics are reported *per frame*: from each frame, we extract the feature vector and use Adaboost to determine the type of the frame, apply the post-processing step and report the number of correctly classified vs. misclassified frames. 

The resulting classification accuracy is 81.75% for the CASME II and 76.71% for the SMIC-E dataset. However, in a real-world application, it will be more appropriate to determine the apex location of the ME and to analyze the frames around this position. In addition, the recognition module takes as input the apex positions of the spotted ME and examines *n* frames centered in the apex locations to determine the type of the ME. 

Based on these conditions, we also report the ME detection results *per emotion:* we compute how many ME were correctly spotted based on the ground truth apex position and the detected apex position. As shown in Section 3.1, the detection post-processing algorithms filters out false positives and determines the apex (or apexes, as several video clips from the SMIC-E database contain more than one expression) of each ME sequence. As opposed to the earlier, *per frame* validation, we consider that the *per emotion* evaluation is more relevant: in the first case, multiple frames (all the frames from the ME interval) are counted as true positives and therefore the metrics can be misleading. Thus, in the *per emotion* validation setup, we consider a ME to be true positive if the absolute distance between the detected apex and the ground truth apex is less than a quarter of the average ME duration expressed in frames:

(9) $$\{\begin{array}{c} TP,\;\left|apex_{gt}-apex_{d}\right|\leq \delta \cdot \tau \\ FP,\;\left|apex_{gt}-apex_{d}\right|>\delta \cdot \tau \end{array}$$

where *TP* stands for True Positive; *FP* stands for False Positive; *apex_gt_* and *apex_d_* are the ground truth and the detected apex position, respectively; *τ* is the average ME duration; and *δ* = 0.25.

The parameters that must be tuned for the ME detection module are related to the post-processing algorithm: the *minMicroSz* and *maxDist.* These values were set to *τ*/4 and *τ*∙2, respectively for the reported results and were determined through trial and error experiments.

The proposed algorithm can detect 80% and 76.92% of the ME on the CASME-II and SMIC-E databases, respectively. The false positive rate is maintained relatively low: 10% for the CASME II dataset and 15.38% for the SMIC-E database. In this context, we refer to the false positive rate as the number incorrectly classified apexes divided by the total number of MEs (apexes) from the testing dataset.

It can be noticed that the algorithm performed better on the CASME-II dataset. One explanation for this could be the length of the ME sequence: the SMIC-E database contains longer video clips, so the detection problem is more challenging. Another cause could be the fact that the CASME-II dataset was captured in a more controlled laboratory environment (using four LED lamps under umbrella reflectors) to avoid the flickering light that usually appears in high speed video sequences.

### 4.2. Micro-Expression Recognition

The proposed micro-analysis framework recognizes only three types of MEs: positive (happiness), negative (disgust, anger, fear, contempt, and sadness) and surprise. We chose this taxonomy for two reasons: (1) some of the MEs are very hard to elicit and not enough training and test data are available; and (2) the distribution between the ME classes in the available datasets is highly imbalanced. Table 2 shows the distribution of the ME classes for the CASME II and the high-speed version of the SMIC-E databases. For example, only 2 fear ME sequences are reported in the literature. Secondly, the samples from the SMIC database are only annotated with these three classes.

The CASME-II dataset also contains some samples (annotated with the “repression” label) that correspond to squelched expressions. These samples were ignored in the training and test process, as they are different from MEs. Squelched expressions [2] usually last longer than MEs and they are not complete in terms of their temporal parameters (onset, apex and offset). MEs occur involuntarily, but in the case of squelched expressions the subject becomes aware of the facial expression, and tries to hide it by rapidly changing his expression (usually with a smile). The MEs from the CASME II dataset labeled with the “others” class were also ignored.

The only parameter that needs to be tuned for the recognition algorithm is the number of frames *n* in which the ME interval is discretized (to make the algorithm invariant to the capturing frame rate). In Table 3, we show the recognition performance using two settings for *n* ϵ {5, 11}.

Better results are obtained using the larger value for *n*. In Table 4, we report the confusion matrices for the recognition problem on the SMIC-E and CASME II dataset for *n* = 11 frames.

In both datasets, there were some confusions between the negative and surprise samples. One possible explanation for this is that the fear expression (classified by the proposed solution as a negative one) is similar to the surprise expression. Surprise raises the eyebrows and lifts the eyelids (FACS action units 1 + 2 + 5), while fear involves raising the eyebrows, then lowering of the brows and finally rise eyelids (FACS action units 1 + 2 + 4 + 5). Thus, there is only a subtle difference between the expression of fear and surprise: the lowering of the brows (action unit 4) which fights against the rising of the eyelids (action unit 5). 

This hypothesis is confirmed by a psychological study [28] performed on a group of South Fore people which were asked to match facial expression to brief stories about emotional events. The subjects selected the correct face in the majorities of cases, but had difficulties in distinguishing fear from surprise. In other words, the same confusion was reported from human subjects.

Regarding the time complexity, the proposed method takes, on average, 6 ms to decide whether a frame contains a ME (detection) and 2 ms to determine the type of the micro-expression (recognition). The system was run on a regular laptop, with a 2.6 GHz Intel Core i7-6700HQ processor.

### 4.3. System Capabilities

**Figure 12:**
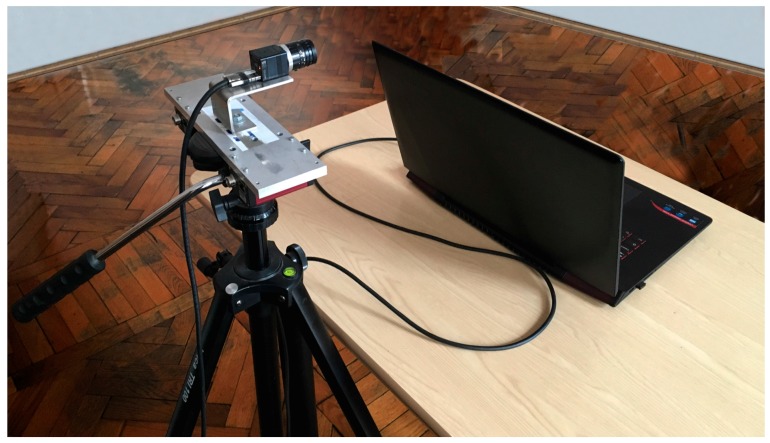
Physical system: the high speed Ximea camera is placed on a tripod and captures high temporal resolution frames of the subject.

The physical system is illustrated in Figure 12.

In Table 5, we report the physical system capabilities under various settings; all the experiments were performed under natural illumination conditions (indoor: Experiments 1–4; or outdoor: Experiments 5–12).

The proposed system can capture facial videos at a frame rate higher than 118 fps in indoor conditions and at a frame rate of more than 200 fps in outdoor conditions. In the indoor case, the exposure time needs to be longer, so the frame rate is lower. We determined the exposure time through trial and error experiments, such that the captured facial image has an optimal brightness value.

The gathering of a quality dataset is a time-consuming task which requires domain specific knowledge (behavioral-psychology, FACS etc.). The ME analysis framework was validated on annotated, publicly available datasets.

### 4.4. Comparison with State of the Art

In this section, we provide a comparison with the recent works from the specialized literature. It should be noted that solely the numerical comparison of the performances is not always relevant, as different testing methodologies and metrics were used. For example, some works use leave-one-sample-out cross validation ([9,10,16]), while, in this manuscript, all the experiments were performed using leave-one-subject-out cross validation. In addition, for the recognition part, some works classify the MEs into more than three classes. 

Most of the works from the literature focused on the problem of ME recognition and only recently, the problem of ME spotting has been addressed. Table 6 shows the ME detection performance compared to the state of the art.

In Table 6, ACC stands for overall accuracy, FPR for False Positive Rate and TPR for True Positive Rate. For the SMIC-E dataset the proposed solution can spot more MEs than [15] with HOOF as features (76.92% vs. 70%), but their solution has the advantage of a lower False Positive Rate (15.38% for the proposed solution vs. 13.5% in [15]). For the CASME-II dataset, the method proposed in [15] using HOOF as features obtains better results (TPR = 82%, FPR = 7%), but the results are close. Therefore, it can be concluded that the ME detection accuracy is comparable when not better to the recent methods published in the literature.

The performance of the recognition module reported to the state of the art is illustrated in Table 7. As mentioned earlier, a direct numerical comparison is not always relevant because on the CASME II dataset some methods classify the MEs with a higher granularity (more ME classes). These methods are marked with a ^+^ symbol in the table.

The majority of the algorithms proposed in the literature split the face into geometrical regions of the same size, extract and concatenate motion descriptors (LBP-TOP, HIGO, Optical Strain, etc.) from each region and finally use a classifier to determine the type of the ME. The main advantage of the proposed solution is its simplicity: the expression type is determined by the simple evaluation of 2D Gaussian probability functions.

The proposed method is outperformed by [15] by 0.8% only on the SMIC-E database. The authors use motion magnification to accentuate the ME movement and extract LBP-TOP features from 36 cells in which the face is divided. Finally, the type of the ME is determined using a support vector machine classifier. However, the proposed method uses simple and fast motion descriptors (absolute differences of images and weighted center computation) from only 10 regions of the face (as opposed to 36). As a future work, we plan to also apply Eulerian video magnification to boost the performance of the recognition algorithm. 

ME analysis has proven to be difficult even for human subjects; at the beginning, even the pioneers of MEs [1] argued that these subtle expressions cannot be spotted in real time. Newer psychological [29] studies showed that the human performance of ME recognition depends on the ME duration. On average, the ME human recognition rate ranges from 51.9% (40 ms ME duration) to 76.6% (300 ms ME duration). 

In [15], the authors also studied the human performance on the high-speed SMIC dataset: the mean accuracy for the subjects was 72.11 ± 7.22%. On the more challenging problem of ME detection and recognition, the mean accuracy of humans is 49.74 ± 8.04% [15]. With a detection rate of 79.23% and a recognition accuracy of 82.59% (averaged CASME II and SMIC-E performances), it can be concluded that the proposed method outperforms human accuracy.

## 5. Conclusions

In this work, we tackled the problem of spontaneous ME analysis. We first designed a ME image acquisition system that does not require rigid position of the subject. The physical system uses two frame acquisition setups: (a) normal angle, full frame, normal frame-rate image acquisition for locating the subject’s face; and (b) a narrow angle, region of interest, high speed acquisition for detection and classification of micro-expressions.

The proposed framework has various applications, such as deceit detection [2], psychology and psychopathology [30] and security [31]. We have proposed novel methods for both ME detection and ME recognition. Both methods rely on the analysis of absolute image differences; we compute the absolute difference between the current frame and its potential apex frame and we divide it with the absolute image difference between the current frame and a neighbor frame. Several regions of the resulting image are analyzed to spot and recognize MEs.

The feature vectors used in both the detection module have a relatively low dimensionality (20d feature vector for ME). Another advantage of the proposed method is that the recognition algorithm is simple and fast, as it only involves the fitting 2D Gaussian functions on the relative center displacements within each region of interest. 

The proposed solution was validated on two publicly available high speed ME databases: CASME II and the high-speed version of the SMIC-E database. With the experiments we performed, we demonstrated that the proposed framework is comparable when not better than the state of the art methods. The main disadvantage of the proposed method is that it was not tested on video sequences where the subjects are allowed to move and rotate their heads freely, as all the ME datasets contain only sequences with non-moving subjects with frontal position. This is the reason the 3D head rotation was not addressed in the current method. The proposed system works well for the frontal use case, but if the subject’s head has a higher vertical head rotation (higher yaw angle), the cell shape should be update so that is compensates this rotation.

As future improvements, we plan to use motion magnification for the recognition to increase the magnitude of the facial expression and boost the algorithm’s performance. Secondly, we also intend to test the proposed method on more natural and interactive scenarios, in which the users are allowed to move their heads freely. All the currently available ME datasets are captured in highly constrained environments (near frontal head position, no head movements, controlled lightning conditions) and have a relatively small number of samples. Using these data, the proposed method did not take into account the 3D head pose information. Finally, in future versions, we intend to also incorporate head tracking information and other facial movement recognition (such as blinks, action unit activations and (macro-) expressions) into the ME detection part.

## Figures and Tables

**Table 1:** ME detection confusion matrices on the CASME II and SMIC-E databases.

		CASME II Dataset		SMIC-E Dataset	
		Predicted		Predicted	
Actual		**ME**	**Non-ME**	**ME**	**Non-ME**
	ME	87.76%	12.23%	77.80%	22.20%
	Non-ME	19.56%	80.43%	23.33%	76.66%

**Table 2:** Distribution of the ME types in the CASME-II and SMIC-E databases.

	CASME II Database		SMIC-E Database	Total
Positive sequences	32		51	83
Surprise sequences	25		42	67
Negative sequence	Fear:	2	71	143
	Disgust:	63		
	Sadness:	7		
Repression sequences *	27		-	27
Other sequences *	99		-	99

* These samples were not used in the training and testing phase of the proposed solution.

**Table 3:** Recognition performance on the CASME II and SMIC-E datasets.

Dataset	Precision	Recall	F1-Score	Accuracy
CASME II; *n* = 5 samples	85%	81%	79%	80.95%
CASME II; *n* = 11 samples	89%	86%	85%	85.71%
SMIC-E; *n* = 5 samples	80%	72%	72%	71.79%
SMIC-E; *n* = 11 samples	86%	79%	80%	79.48%

**Table 4:** Recognition confusion matrices on the CASME II and SMIC-E datasets.

		CASME II Dataset			SMIC-E Dataset		
		Predicted			Predicted		
Actual		**Positive**	**Negative**	**Surprise**	**Positive**	**Negative**	**Surprise**
	Positive	100%	0	0	75%	8.33%	16.66%
	Negative	0	100%	0	0	70.59%	29.41%
	Surprise	0	37.5%	62.5%	0	0	100%

**Table 5:** Physical system performance under various illumination setting.

Experiment	Exposure Time (ms)	Frames per Second (fps)	Distance to Subject (cm)	Light Intensity (Lux)	Image Size (Pixels)
1	7	118	80	770	654 × 654
2	7	138.5	100	770	458 × 458
3	7	138	120	770	386 × 386
4	7	139	200	770	246 × 246
5	5	179	80	1100	497 × 497
6	5	198	100	1100	404 × 404
7	5	198	120	1100	360 × 360
8	5	199	200	1100	239 × 239
9	4	189	80	1300	542 × 542
10	4	243	100	1300	423 × 423
11	4	249	120	1300	358 × 358
12	4	249	200	1300	255 × 255

**Table 6:** ME detection performance—comparison with state of the art.

Method	Features	Performance	
		CASME II	SMIC
[14]	Optical strain, LBP-TOP	-	74.52% *
[15]	HOOF	TPR = 82% **	TPR = 70%
		FPR = 7% **	FPR = 13.5%
[15]	LBP	TPR = 78% **	TPR = 85% **
		FPR = 45% **	FPR = 5% **
[20]	HOOF	ACC = 80%	-
[9]	LBP-TOP	-	ACC =74.3% *
Proposed solution	Frame differences	ACC = 81.75%	ACC = 76.71%

* The methods were evaluated on the original SMIC database and not on the extended version; ** Values determined from the ROC (receiver operating characteristic) curve plots.

**Table 7:** ME recognition performance—comparison with state of the art.

Method	Features	Classifier	Accuracy	
			CASME II	SMIC
[14]	Optical strain, LBP-TOP	SVM	ACC = 63.16% ^+^	ACC = 58.15%
[15]	HIGO-YOT	SVM	ACC = 57.09%	ACC = 78.87%
	HIGO-TOP	SVM	ACC = 55.87%	ACC = 80.28%
[18]	CNN + RNN	RNN	ACC = 59.47%	-
[10]	LBP-TOP	SVM	-	ACC = 48.80%
[16]	Optical strain	SVM	ACC = 50.00% ^+^	ACC = 66.40%
Proposed solution	Relative center displacement		ACC = 85.71%	ACC = 79.48%

^+^ CASME II recognition with more than three classes.

## Supplementary Materials

The following are available online at http://www.mdpi.com/1424-8220/17/12/2913/s1, Video S1: Demo of the micro-expression detection and recognition process. In the video, the ground truth annotation for the ME (onset-apex-offset) is marked with a yellow circle in the top left of the video. The detected ME interval is marked with a yellow square to the right of the displayed circle.
